# An update on oxysterol biochemistry: New discoveries in lipidomics

**DOI:** 10.1016/j.bbrc.2018.02.019

**Published:** 2018-10-07

**Authors:** William J. Griffiths, Yuqin Wang

**Affiliations:** Institute of Life Science, Swansea University Medical School, Singleton Park, Swansea SA2 8PP, UK

**Keywords:** Cholesterol, Sterol, LC-MS, Cancer, Immunity, Cytochrome P450, Hydroxysteroid dehydrogenase

## Abstract

Oxysterols are oxidised derivatives of cholesterol or its precursors post lanosterol. They are intermediates in the biosynthesis of bile acids, steroid hormones and 1,25-dihydroxyvitamin D_3._ Although often considered as metabolic intermediates there is a growing body of evidence that many oxysterols are bioactive and their absence or excess may be part of the cause of a disease phenotype. Using global lipidomics approaches oxysterols are underrepresented encouraging the development of targeted approaches. In this article, we discuss recent discoveries important in oxysterol biochemistry and some of the targeted lipidomic approaches used to make these discoveries.

## Highlights

• Oxysterols can regulate both the innate and adaptive immune systems.
• Oxysterols can be tumour suppressors and on cometabolites.
• Oxysterols can inhibit or activate the Hh signalling pathway.

## Graphical abstract

**Figure:**
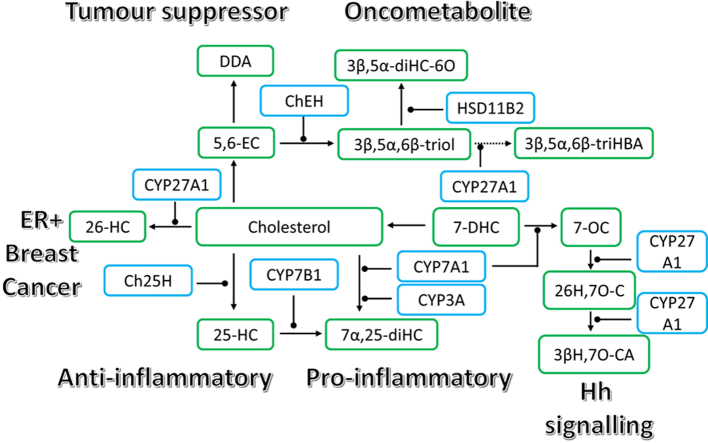

## 1 Introduction

Oxysterols came to prominence in the late 1970's with the *oxysterol hypothesis* which proposed that the suppressive effect of cholesterol on its own synthesis is mediated through oxysterols not by cholesterol itself [1]. This has proved to be only partly true, as cholesterol homeostasis in cells is modulated through oxysterols, side-chain oxysterols inhibiting the SREBP2 (sterol regulatory element-binding protein 2) pathway and also activating liver X receptors (LXRs) [2,3], although cholesterol itself is in fact the major regulator of its own synthesis through binding to SCAP (SREBP cleavage activating protein) and preventing transport of SERBP2 from the endoplasmic reticulum for processing to its active form as a transcription factor for genes of the cholesterol biosynthesis pathway [4]. In recent years oxysterols have been shown to have important functions in immunology [[5], [6], [7], [8], [9], [10], [11], [12], [13]], development [14,15] and cancer [[16], [17], [18], [19], [20]]. This has stimulated wide-spread interest in their analysis using lipidomics technology [21,22]. Although mostly thought of as oxidised forms of cholesterols, oxysterols can also be formed from precursors of cholesterol greatly widening the range of molecules required to be analysed in a lipidomic study.

## 2 Mass spectrometry-based technologies

Oxysterols tend not to be observed in global lipidomic analysis, whether shot-gun based electrospray ionisation – mass spectrometry (ESI-MS) or liquid chromatography (LC)-MS based. This is because of their comparatively low-abundance and poor ionisation characteristics. However, methods have been developed for shot-gun ESI – tandem MS (MS/MS) [23] and LC-MS/MS analysis [21,22]. Gas chromatography (GC)-MS also provides an excellent method for oxysterol and sterol analysis [24], but is less favoured in lipidomics laboratories.

### 2.1 Targeted LC-MS/MS analysis

Russell and McDonald and co-workers [21,22] have developed LC-MS/MS lipidomics protocols for oxysterol analysis based on the classical sample preparation method of Dzeletovic et al. [24] and multiple reaction monitoring (MRM) using a triple quadrupole mass spectrometer exploiting [M + NH_4_]^+^ adducts for fragmentation. They have made the largest study to date in terms of sample numbers, analysing 3230 serum samples for 60 sterols [22]. The McDonald and Russell method is the template on which many other LC-MS/MS studies have been made [25]. An inherent drawback, however, of targeted approaches is that only targeted oxysterol are detected and unexpected ones are missed.

### 2.2 LC-MS/MS with derivatisation

The concept of targeted oxysterol analysis has been extended with the use of derivatisation methods to enhance sensitivity. Derivatisation of hydroxy groups to dimethylglycine or *N,N*-dimethylaminobutyrate esters has recently gained popularity for the diagnosis of Niemann Pick (NP) type B and C disease where 7-oxocholesterol (7-OC) and cholestane-3β,5α,6β-triol (3β,5α,6β-triol, see Supplementary Table for a list of common and systematic names and abbreviations and Supplementary Figures 1 & 2 for structures) are elevated in plasma of NP patients [26,27], while derivatisation to picolinate esters has proved successful for analysis of both sterols and oxysterols [28]. “Primary” oxystrols have a 3β-hydroxy group, however, once 7α-hydroxylated, the 3β-hydroxy-5-ene function can be oxidised to a 3-oxo-4-ene by the enzyme hydroxysteroid dehydrogenase (HSD) 3B7 (Supplementary Figure 1). The 3-oxo group is readily derivatised by hydrazine reagents, e.g. quaternary nitrogen containing Girard hydrazines, (hydrazinocarbonylmethyl)trimethylammonium chloride, GT or 1-(hydrazinocarbonylmethyl)pyridinium chloride, GP [29], or hydroxylamine reagents, e.g. *O*-(3-trimethylammoniumpropyl) hydroxylamine bromide [30], to enhance sensitivity during plasma analysis, valuable for the identification of patients with inborn errors of metabolism. The 3β-hydroxy-5-ene function found in “primary” oxysterols can be oxidised *ex vivo* by bacterial cholesterol oxidase to the 3-oxo-4-ene function [31], this has been exploited with subsequent Girard derivatisation, using GP or GT, in numerous studies to enhance the sensitivity of oxysterol analysis [8,10,19,[32], [33], [34]]. Despite advantages, derivatisation technology is often criticised for being laborious and difficult to automate.

## 3 Lipidomics and oxysterol biochemistry

### 3.1 Cholesterol 25-hydroxylase, 25-hydroxycholesterol and the immune system

**Fig. 1:**
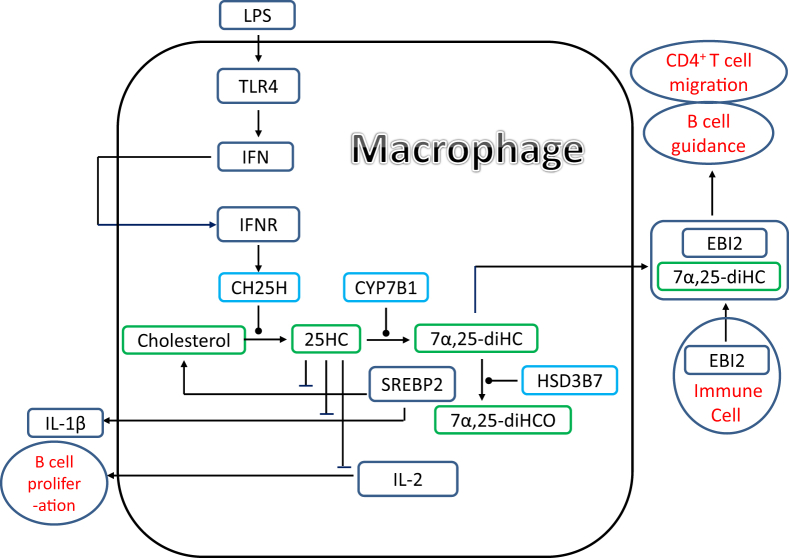
Schematic representation of the regulation of immune responses by 25-HC and 7α,25-diHC.

Cholesterol 25-hydroxylase (CH25H in man and Ch25h in mouse) is the enzyme which oxidises cholesterol to 25-hydroxycholesterol (25-HC) (Supplementary Figure 1). CH25H is not a cytochrome P450 (CYP), but a member of a family of enzymes that utilize diiron cofactors to catalyse hydroxylation. Some CYP enzymes also have some 25-hydroxylase activity, but often secondary to their main catalytic functions [35]. In a major LC-MS lipidomic study investigating the effect of Kdo2-lipid A, the active component of an inflammatory lipopolysaccharide (LPS), on the mouse macrophage cell line RAW264.7, Dennis et al. found 25-HC to be elevated in abundance, correlating with a 4-fold increase in *Ch25h* mRNA [36]. The mRNA increase was 15-fold in bone marrow derived macrophages. In a parallel study, Bauman et al. showed that stimulation of macrophage Toll-like receptor 4 (TLR4) induced *Ch25h* expression and 25-HC synthesis and that 25-HC suppressed interleukin-2 (IL-2) mediated stimulation of B-cell proliferation, repressed activation induced cytidine deaminase (AID) expression, and blocked class switch recombination, leading to markedly decreased IgA production [5] (Fig. 1). Suppression of IgA class switching in B cells in response to TLR activation provides a mechanism for local and systemic negative regulation of the adaptive immune response by the innate immune system [5]. *Ch25h* is an interferon (IFN) regulated gene and upon viral infection, or IFN-stimulation, 25-HC is synthesised by macrophages and acts as a potent paracrine inhibitor of viral infection demonstrating a role in the innate immune pathway [8].

Reboldi et al. have shown that activated macrophages from the *Ch25h*^−/-^ mouse overproduce inflammatory IL-1 family cytokines [9]. They proposed that 25-HC represses *Il1b* transcription and repressed IL-1 activating inflammasomes by repressing the SREBP2 pathway [9]. Reboldi et al. also found that *Ch25h*^−/-^ mice exhibited increased sensitivity to septic shock, exacerbated experimental autoimmune encephalomyelitis (EAE), a mouse model for multiple sclerosis, and a stronger ability to repress bacterial growth. They concluded that 25-HC is a mediator in the negative feedback pathway of IFN-signalling on IL1-family cytokine production and inflammasome activity. In agreement with the multiple sclerosis mouse model, Crick et al. in a LC-MS based sterolomic study exploiting GP-derivatisation found reduced plasma levels of 25-HC in plasma of multiple sclerosis patients [34].

Further understanding of the role of 25-HC in the inflammatory system comes from the work of Dang et al. [12], who confirmed that type 1 IFN restrains 1L-1β driven inflammation in macrophages by up-regulating *Ch25h* and 25-HC and repressing SREBP2 driven cholesterol synthesis. In the absence of *Ch25h,* cholesterol overload triggers mitochondrial DNA release and activation of AIM2 (absent in melanoma 2) inflammasomes in activated macrophages [12]. The inflammasome promotes the maturation of the inflammatory cytokines e.g. IL-1β by recruitment and activation of caspase-1 that processes IL-1β into its active form. Consequently, in *Ch25h*^−/-^ macrophages there is an exaggerated response to pathogen derived activators.

There is just one report of CH25H deficiency in man. Goenka et al. reported on five unrelated infants born to consanguineous parents showing homozygous deletions of *CH25H* and the adjacent gene *LIPA* (lipase A, lysosomal acid type) in all five children [37]. *LIPA* encodes the enzyme lysosomal acid lipase, deficiency in which leads to Wolman disease in infants, a lysosomal storage disease characterised by accumulation of cholesterol esters. Neonatal BCG (Bacillus Calmette–Guérin) vaccination against *Mycobacterium tuberculosis* infection was performed on four of the children and in three local BCG abscesses developed. Otherwise, none of the children had any other history of prolonged, unusual or recurrent infections [37]. It is tempting to speculate that the absence of macrophage derived 25-HC leads to an exaggerated response to the BCG vaccination and abscess.

In contrast to the findings of Reboldi et al. [9], Chalmin et al. found that deletion of *Ch25h* (*Ch25h*^−/−^) attenuated EAE [11], limiting the trafficking of CD4^+^ T cells to the central nervous system. Chamlin et al. explained this by proposing that 7α,25-dihydroxycholesterol (7α,25-diHC), a downstream metabolite of 25-HC, promotes the migration of activated proinflammatory T cells up a gradient of 7α,25-diHC by binding to the G protein-coupled receptor (GPCR) Epstein-Barr virus-induced gene 2 (EBI2, GPR183, Fig. 1) [11].

### 3.2 EBI2 and 7α,25-diHC in immunity

EBI2 regulates the positioning of immune cells in secondary lymphoid organs (sites of lymphocyte activation by antigens) and polymorphisms in the receptor have been associated with inflammatory autoimmune diseases. Hannedouche et al. purified the ligand for EBI2 by exploiting a bioactivity assay and sheep sepsis model [7]. By high resolution ESI-MS the ligand was identified as a cholestentriol. Following further purification and NMR analysis, the ligand was identified as 7α,25-diHC. In a study published at the same time, Liu et al. confirmed the EBI2 ligand as 7α,25-diHC [6]. The positional isomer 7α,(25R)26-dihydroxycholesterol (7α,26-diHC) was shown to have similar activation capacity towards EBI2 and both isomers act as chemoattractants for immune cells expressing EBI2 [7].

7α,25-diHC can be synthesised by multiple metabolic pathway (Supplementary Figure 1). One route is via 25-hydroxylation of cholesterol by CH25H followed by 7α-hydroxylation by CYP7B1. CYP3A enzymes also have sterol 25-hydroxylation activity as do CYP27A1 and CYP46A1 [35]. 7α,25-diHC can alternatively be formed via 7α-hydroxylation of cholesterol by CYP7A1 followed by 25-hydroxylation of 7α-hydroxycholesterol (7α-HC) to give 7α,25-diHC. By whatever route it may be formed 7α,25-diHC is inactivated by HSD3B7. In *Ch25h*^−/−^ mice 7α,25-diHC was found to be at, or below, the detection limit in spleen and lymph node tissue extracts, but in wildtype mice was clearly detectable following LPS challenge indicating that 7α,25-diHC is generated in the spleen in a CH25H-dependent manner [7]. Interestingly, Hannedouche at al found that similar to *EBI2*^−/−^ mice, *Ch25h*^−/−^ mice fail to position activated B cells within the spleen to the outer follicle and mount a reduced plasma cell response after an immune challenge [7]. This supports the hypothesis that 7α,25-diHC is generated in a CH25H dependent manner and has a proinflammatory function.

In a recent study, Wanke et al. confirmed that EBI2 in combination with 7α,25-diHC direct immune cell localisation in secondary lymphoid organs [13]. They found that EBI2 is expressed by Th17 cells in inflammation and that EBI2 promotes early CNS infiltration in passive EAE [13]. In a targeted lipidomic study for oxysterols they found, using LC-MS/MS methodology [25], that not only 25-HC and 7α,25-diHC were of enhanced abundance in spinal cord of mice with EAE, but also 7α,26-diHC and 7α,24-dihydroxycholesterol (7α,24-diHC), although 24-hydroxycholesterol (24-HC) and 26-hydroxycholesterol (26-HC) were less abundant than in naïve mice [13]. In EAE mice expression of both *Ch25h and Cyp7b1* were up-regulated, but *Hsd3b7* was reduced. Interestingly, microglia were found to express *Ch25h* during EAE, while *Cyp7b1* was expressed in infiltrating lymphocytes and monocytes. *Hsd3b7* was expressed by both cellular populations. *Ch25h* was not expressed in microglia from the healthy CNS [13].

In summary, there is conflicting evidence regarding the role of CH25H and 25-HC in autoimmunity. Data from Reboldi et al. suggests that *Ch25h*^−/−^ mice experience exacerbated EAE [9] and Dang et al. have shown that 1L-1β driven inflammation in macrophages is restrained by up-regulating *Ch25h* and 25-HC [12]. On the other hand, Chalmin et al. found that deletion of *Ch25h* attenuated EAE [11], while Wanke et al. found that *Ch25h* and *Cyp7b1* were up-regulated in the CNS of EAE mice [13].

### 3.3 Oxysterols and hedgehog signalling

The hedgehog (Hh) signalling pathway is essential for animal development. It provides a communication system between cells of adult and of developing tissue and regulates cell proliferation, stem cell maintenance and fate specification. Hh signalling can be misactivated to cause cancer.

**Fig. 2:**
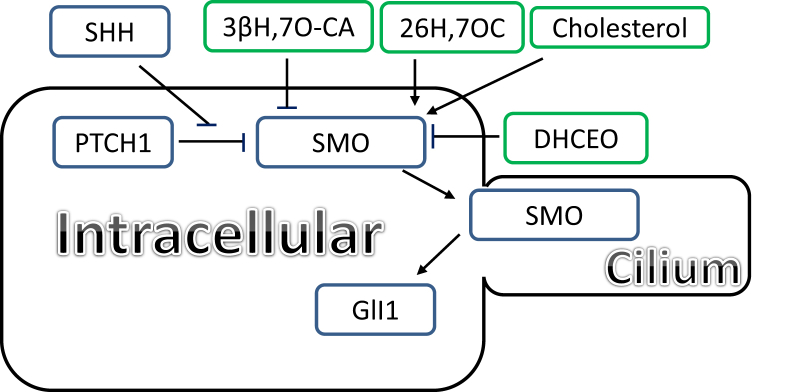
Schematic representation of the role of oxysterols in the Hh signalling pathway.

The phenotype of congenital Hh deficiency is similar to that found in some inborn errors of cholesterol biosynthesis e.g. lathosterolosis, desmosterolosis and Smith-Lemli-Opitz syndrome (SLOS), where cholesterol biosynthesis is reduced and its precursors, lathosterol, desmosterol and 7-dehydrocholesterol (7-DHC) accumulate in the respective diseases (Supplementary Figure 2) [38]. Three protein families are critically involved in Hh signalling. Of these, the Hh ligands, sonic hedgehog (SHH), indian hedgehog (IHH) and desert hedgehog (DHH), are modified with cholesterol at the *C*-terminus. In vertebrates SHH is the most well studied and is secreted into the extracellular space where it binds to the membrane associated protein Patched 1 (PTCH1). PTCH1 indirectly inhibits transport of the GPCR Smoothened (SMO), a 7-transmembrane domain (TMD) protein with an extracellular cysteine rich domain (CRD), to primary cilium, antenna that project from the surface of most cells. When SHH binds to PTCH1 repression of SMO is relieved and SMO enters the cilium where a second activation step initiates down-stream signalling through GLI1 transcription factors which regulate developmental patterning (Fig. 2) [39]. Inhibition of SMO by PTCH1 can be overcome by cholesterol and oxysterols in the absence of SHH. Cholesterol has been found to be not just necessary but also sufficient to activate signalling by the Hh pathway, binding to SMO at its CRD [40,41], the same binding site as some oxysterols. Two oxysterols of elevated abundance in SLOS and derived from 7-DHC [42], (25R)26-hydroxy-7-oxocholesterol (26H,7O-C) and 25-hydroxy-7-oxocholesterol (25H,7-OC) have been found to activate the Hh signalling pathway via binding to the CRD of SMO [14], while the downstream metabolite of 26H,7O-C, 3β-hydroxy-7-oxoxholest-5-en-(25R)26-oic acid (3βH,7O-CA, Supplementary Figure S2) inhibits Hh signalling in the presence of SHH ligand, again by binding to the CRD of SMO [42]. SLOS, which results from 7-dehydrocholesterol reductase (DHCR7) deficiency, phenocopies Hh deficiency, and we speculate that dysmorphology observed in SLOS is a consequence of dysregulated Hh signalling. Another oxysterol, 3β,5α-dihydroxycholest-7-en-6-one (DHCEO), like 25H,7O-C, 26H,7O-C and 3βH,7O-CA, derived from 7-DHC, also inhibits Hh signalling by binding to SMO, but at a site distinct from other oxysterols [15]. Unlike, 25H,7-OC, 26H,7-OC and 3βH,7O-CA which are formed enzymatically from 7-DHC, DHCEO is formed from 7-DHC via free radical reactions.

### 3.4 Oxysterols and cancer

**Fig. 3:**
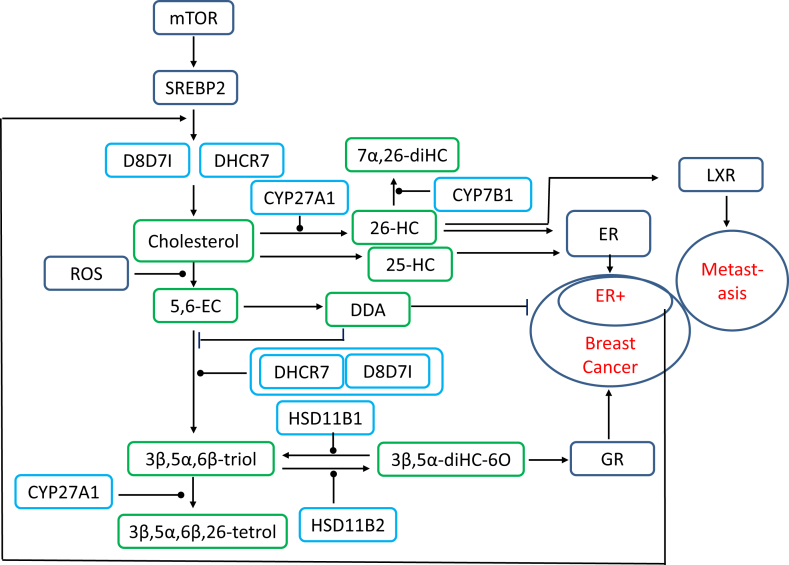
Schematic representation of the involvement of oxysterols in cancer.

Recent studies have uncovered important links between oxysterols and breast cancer. Dendgrogenin A (DDA), a histamine conjugate of 5α,6-epoxycholesterol (5α,6-EC, Supplementary Figure 1), was first identified in mouse brain tissue by LC combined with off-line MS and MS with multistage fragmentation (MS^n^) and comparison to an authentic standard [18]. DDA was not detected in cancer cell lines and was less abundant in human breast tumours compared to normal tissue. DDA triggered tumour re-differentiation and growth control in mice, improving animal survival [18]. The precursor of DDA, 5α,6-EC, and its isomer 5β,6-EC, can alternatively be converted enzymatically by cholesterol expoxide hydrolase (ChEH) to 3β,5α,6β-triol. DDA is an inhibitor of ChEH, which itself is a heterodimer of two enzymes of the cholesterol biosynthesis pathway, DHCR7 and 3β-hydroxysteroid-Δ-8,7-sterol isomerase (D8D7I, Fig. 3) [18]. 3β,5α,6β-triol can be metabolised to the bile acid 3β,5α,6β-trihydroxycholanoic acid (3β,5α,6β-triHBA) [43,44] through metabolism by CYP27A1 followed by side-chain shortening by peroxisomal enzymes, or alternatively be oxidised at C-6 to 3β,5α-dihydroxycholestan-6-one (3β,5α-diHC-6O) by HSD11B2 [20]. 3β,5α-diHC-6O was found to stimulate breast cancer cell proliferation both *in vivo* and *in vitro* in estrogen receptor positive (ER+) and ER negative (ER-) cells and tissues [20]. In a targeted analysis utilising LC separation off line with chemical ionisation -MS 3β,5α-diHC-6O was identified in breast cancer tissue at higher levels than in adjacent normal tissue. Interestingly, breast cancer samples showed greater expression of the two enzymes required for 3β,5α-diHC-6O formation from 5,6-EC, i.e. ChEH and HSD11B2, than normal tissue and in analysis of breast cancer mRNA databases, overexpression of HSD11B2 and ChEH correlated with a higher risk of patient death [20]. 3β,5α-diHC-6O was found to be a ligand to the glucocorticoid receptor (GR), and high expression of the GR correlates with poor therapeutic response or prognosis in many solid tumours [20]. 5α,6-EC, the precursor of DDA, along with its isomer 5β,6-EC sit at the fulcrum of tumour progression. Conjugation with histamine by an enzyme still to be identified will lead to the tumour suppressor DDA, alternatively, hydrolysis by ChEH to 3β,5α,6β-triol then oxidation by HSD11B2 will lead to the oncometabolite 3β,5α-diHC-6O. Metabolism of 3β,5α,6β-triol to 3β,5α-diHC-6O can be diverted by CYP27A1 oxidation to cholestane-3β,5α,6β, (25R)26-tetrol (3β,5α,6β,26-tetrol) and ultimately to the bile acid 3β,5α,6β-triHBA. In this case expression of CYP27A1 in breast cancer tissue would lead to the deactivation of 3β,5α,6β-triol, however, it should be noted that the biological activity of 3β,5α,6β,26-tetrol and its metabolites have yet to be explored.

A link between breast cancer and CYP27A1, in this case to generate an oncogenic metabolite, 26-HC, (also known as 27-hydroxycholesterol) was established by two group, Wu et al. [16] and Nelson et al. [17], who published their data almost simultaneously. 26-HC is a known selective estrogen receptor modulator (SERM), and using the LC-MS/MS method of McDonald et al. [21], Wu et al. showed that the tumour content of 26-HC was elevated compared to normal tissue from ER + breast cancer patients and that normal tissue from these patients had a higher content of 26-HC than tissue from controls [16]. 26-HC was shown to stimulate MCF-7 cell xenograft growth in mice [16]. Integration by Wu et al. of the Cancer Genome Atlas found that *CYP27A1* expression was similar in normal breast and ER + tumours but *CYP7B1* expression was decreased by 50% in ER + tumours compared with normal breast tissue [16]. CYP7B1 is the oxysterol 7α-hydroxylase that metabolises 26-HC to 7α,26-diHC. Interestingly, serum analysis of women with ER + breast cancer and cancer free subjects showed that at presentation the mean 26-HC levels were not significantly different between the two groups, indicating that local metabolism must account for elevated 26-HC in breast cancer tissue.

Nelson et al. also showed that 26-HC was increased in ER + mouse models of breast cancer and that in human breast cancer tissue CYP27A1 protein expression correlated with tumour grade, increased expression of this enzyme was observed in higher grade tumours [17]. This study also demonstrated LXR-dependent metastasis in mouse models of breast cancer; significantly 26-HC is a ligand to LXR [17].

While tamoxifen, a SERM, which also binds to and inhibits ChEH, is prescribed to both pre- and post-menopausal women with ER + breast cancer, aromatase inhibitors are used to treat postmenopausal women with ER + breast cancer. Despite endocrine therapy, many patients relapse because of adaptive changes associated with resistance to aromatase inhibitors. Long-term estrogen deprivation (LTED) of ER + cell lines provide a model of relapse to aromatase inhibitors and Simigdala et al. have shown using proteomic and transcriptomic analysis that the cholesterol biosynthesis pathway is upregulated in ER + LTED but not ER- LTED cell lines [19]. Interestingly, in light of the study by Voisin et al. [20], D8D7I was particularly upregulated in proteomic analysis of the ER + breast cancer cell line MCF7 LTED cells. Using LC-MS/MS with GT derivatisation Simigdala et al. showed a significant increase in the concentration of 25-HC but not 26-HC in MCF7 LTED cells compared to the parental cell line [19]. Like, 26-HC, 25-HC increased ER mediated transcription of ER regulated genes in MCF7 LTED but not ER- LTED cells. In terms of absolute cell number, addition of 25-HC or 26-HC to MCF7 LTED cells resulted in a small but significant increase. In addition, the antiproliferative effect of fulvestrant, a selective estrogen receptor degrader, in MCF7 LTED cells was reduced by addition of 25-HC or 26-HC [19]. In summary, this data indicates that 25-HC, like 26-HC, a SERM, plays a role in the resistance of ER + breast cancer to aromatase therapy.

Although the serum concentration of 26-HC does not appear to differ between ER + breast cancer patients and controls, the possibility exists that evidence for an increased local production becomes lost by analysing the bulk medium. Exosomes are extracellular vesicles released by exocytosis, they are rich in lipids and are released by cancer cells in greater numbers than by healthy cells. This led Roberg-Larsen et al. to investigate using capillary LC – MS/MS with GT derivatisation the oxysterol content of exosomes. They found increased 26-HC in exosomes from ER + MCF7 cells compared to ER- breast cancer cell lines or other non-cancer cell lines. They concluded that oxysterol profiling of cancer cells may have diagnostic value [33].

In summary, recent studies have revealed the importance of oxysterols in health and disease. While 25-HC generated by CH25H in macrophages appears to have an anti-inflammatory function, its CYP7B1 metabolite 7α,25-diHC appears to be pro-inflammatory. CYP27A1 metabolises cholesterol to the ER + breast cancer oncometabolite 26-HC, but CYP27A1 also provides a deactivation rout for 3β,5α,6β-triol diverting its metabolism from the oncometabolite 3β,5α-diHC-6O to bile acid biosynthesis. CYP27A1 is also critical for production of the Hh pathway activator 26H,7O-C and inhibitor 3βH,7O-CA. In healthy tissue the production and removal of oxysterols is exquisitely regulated. Dysregulation may lead to disease.
