# Supplementary material for: An update on oxysterol biochemistry: New discoveries in lipidomics
Source: Biochem Biophys Res Commun. 2018 Oct 7;504(3):617–22. doi: 10.1016/j.bbrc.2018.02.019 (PMC6381446; doi:10.1016/j.bbrc.2018.02.019)
Supplement: Online data [file mmc2.pptx]

## Slide 1
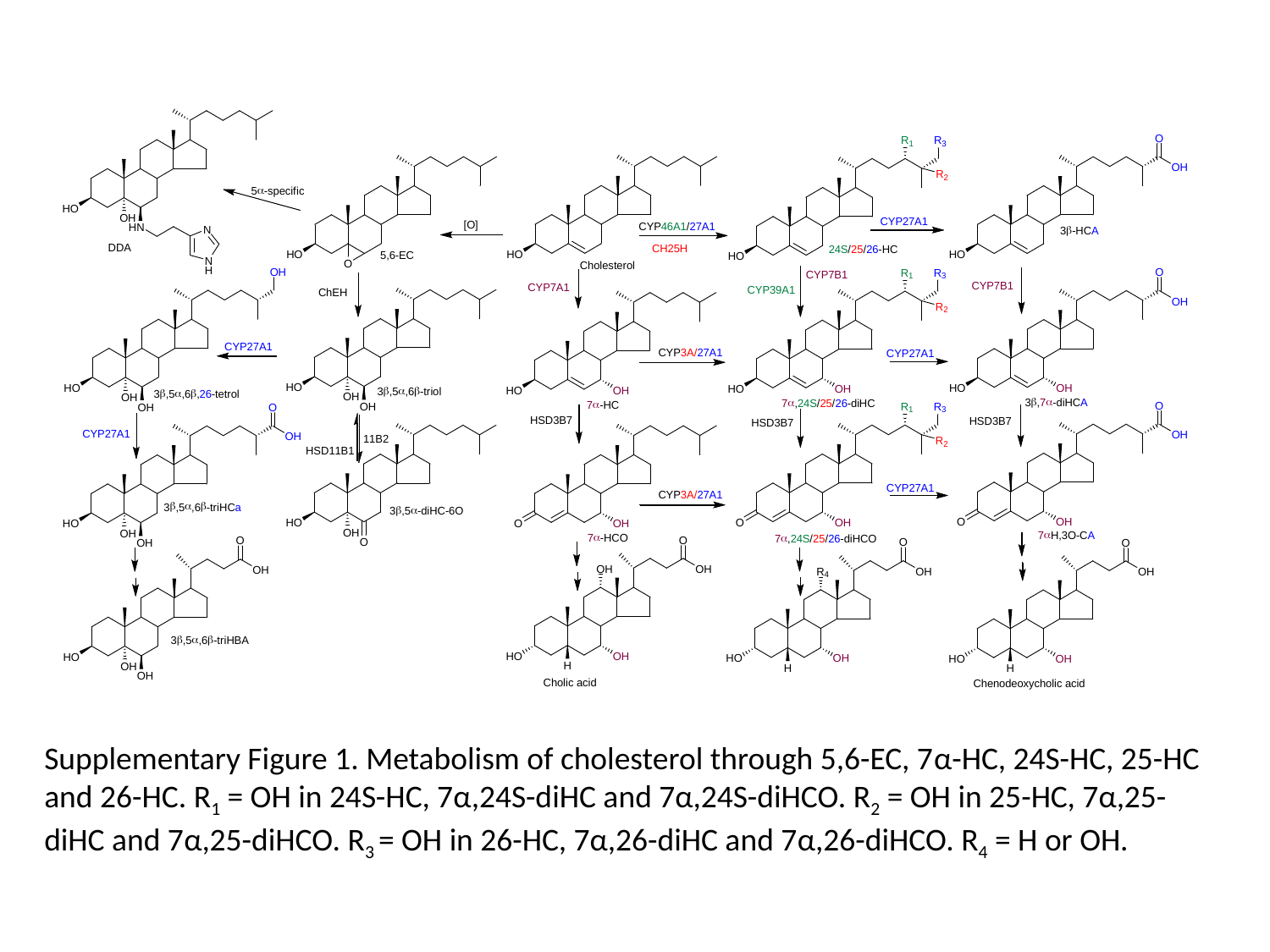

Supplementary Figure 1. Metabolism of cholesterol through 5,6-EC, 7α-HC, 24S-HC, 25-HC and 26-HC. R1 = OH in 24S-HC, 7α,24S-diHC and 7α,24S-diHCO. R2 = OH in 25-HC, 7α,25-diHC and 7α,25-diHCO. R3 = OH in 26-HC, 7α,26-diHC and 7α,26-diHCO. R4 = H or OH.

## Slide 2
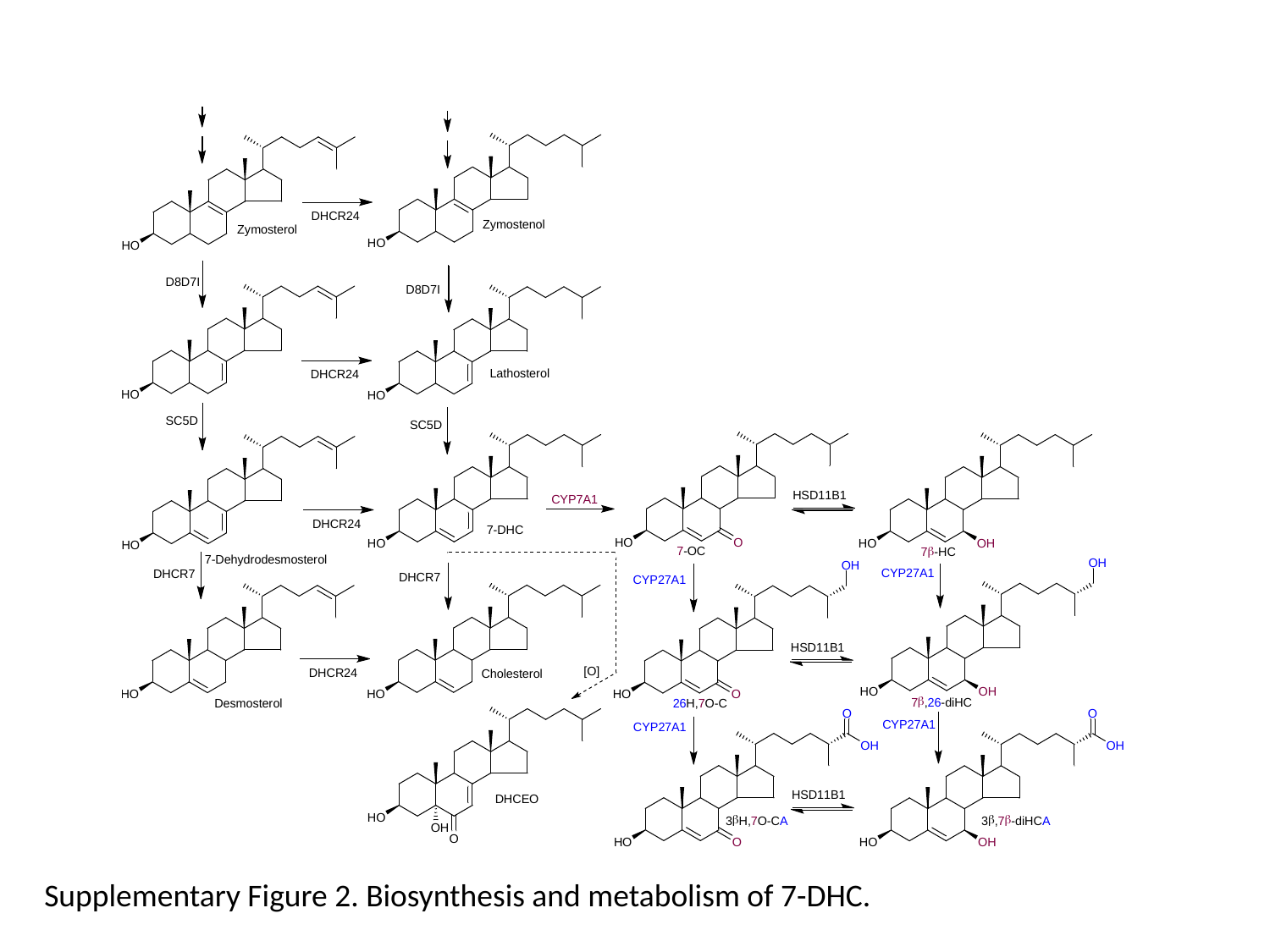

Supplementary Figure 2. Biosynthesis and metabolism of 7-DHC.
