# Supplementary material for: An update on oxysterol biochemistry: New discoveries in lipidomics
Source: Biochem Biophys Res Commun. 2018 Oct 7;504(3):617–22. doi: 10.1016/j.bbrc.2018.02.019 (PMC6381446; doi:10.1016/j.bbrc.2018.02.019)
Supplement: Online data [file mmc3.docx]

Swansea University has patented the Girard hydrazine derivatisation technology described in this paper, US9851368 B2. Swansea Innovations Ltd have licensed the Girard hydrazine derivatisation technology described in this paper to Avanti Polar Lipids Inc and Cayman Chemical Company.
